# RNA-Dependent RNA Polymerase (NIb) of the Potyviruses Is an Avirulence Factor for the Broad-Spectrum Resistance Gene *Pvr4* in *Capsicum annuum* cv. CM334

**DOI:** 10.1371/journal.pone.0119639

**Published:** 2015-03-11

**Authors:** Saet-Byul Kim, Hye-Young Lee, Seungyeon Seo, Joo Hyun Lee, Doil Choi

**Affiliations:** Department of Plant Science, Plant Genomics and Breeding Institute, Seoul National University, Seoul, Korea; Agriculture and Agri-Food Canada, CANADA

## Abstract

Potyviruses are one of the most destructive viral pathogens of Solanaceae plants. In *Capsicum annuum* landrace CM334, a broad-spectrum gene, *Pvr4* is known to be involved in resistance against multiple potyviruses, including *Pepper mottle virus* (PepMoV), *Pepper severe mosaic virus* (PepSMV), and *Potato virus Y* (PVY). However, a potyvirus avirulence factor against Pvr4 has not been identified. To identify the avirulence factor corresponding to Pvr4 in potyviruses, we performed *Agrobacterium*-mediated transient expressions of potyvirus protein coding regions in potyvirus-resistant (*Pvr4*) and -susceptible (*pvr4*) pepper plants. Hypersensitive response (HR) was observed only when a RNA-dependent RNA polymerase (NIb) of PepMoV, PepSMV, or PVY was expressed in *Pvr4*-bearing pepper leaves in a genotype-specific manner. In contrast, HR was not observed when the NIb of *Tobacco etch virus* (TEV), a virulent potyvirus, was expressed in *Pvr4*-bearing pepper leaves. Our results clearly demonstrate that NIbs of PepMoV, PepSMV, and PVY serve as avirulence factors for Pvr4 in pepper plants.

## Introduction

Potyviruses belong to the family *Potyviridae* which represents the largest plant viruses, and severely affect the production of economically important crops. Several members of the genus *Potyvirus* including *pepper mottle virus* (PepMoV), *pepper severe mosaic virus* (PepSMV), *potato virus Y* (PVY) and *tobacco etch virus* (TEV) have a wide range of hosts such as potato, pepper, and tomato in Solanaceae plants [1]. The genome of potyviruses is composed of a single-stranded RNA with a length of ∼9.7 kb, which covalently links with a viral-encoded protein (VPg) at its 5’-end and contains a 3’polyadenylated tail. All members of potyviruses encode two polyproteins, a larger polyprotein of about 3,000 amino acids and the shorter one translated from a 2+ frameshift in the P3 coding region [2]. These polyproteins are cleaved by viral proteases subsequently generating eleven mature proteins [3].

To date, functions of PVY viral proteins are the most well studied among potyviruses in response mechanisms against plant host factors to trigger the plant immune system [2,4–8]. For example, PVY VPg interacts with a recessive resistance protein, pvr2 in pepper which is also known as a member of eukaryotic initiation factor 4E (elF4E) [9]. Another PVY viral protein, HC-Pro is known to function broadly in potato and tobacco by interacting with elF4E and its elFiso4E [10], and is also involved in HR-like cell death in potato by responding to resistance genes called *NC*_tbr_, *NC*_spl_ and *Ny*_tbr_ [7]. A PVY protease, NIa protease (also called NIaPro) was found to be required for *Ry*-mediated resistance of potato against PVY [5]. While these PVY viral proteins have structural analogy with other potyvirus proteins, they do not always function similar. For instance, a PepMoV NIaPro which exhibits 63.5% identity in sequence with a PVY NIaPro showed HR in *Ry*-mediated resistance; whereas, a TEV NIaPro failed to induce HR although it shares 45.9% identity with the PVY NIaPro [5].

PepMoV was first reported as an atypical pepper isolate of PVY [11], is known to cause a serious disease in pepper [12]. However, functions of PepMoV-encoded proteins mostly remain unknown.

The completion of the pepper genome sequencing project using *Capsicum annuum* landrace CM334 (hereafter CM334) provides a tremendous amount of information and facilitates characterization of multiple disease resistance genes in pepper [13]. CM334 contains a single dominant resistance gene, referred as *Pvr4*, which confers resistance against all strains of PepMoV, PepSMV, and PVY, but not to TEV [6,14–18]. The *Pvr4*-mediated resistance in pepper plants exhibits extreme resistance or HR to multiple potyviruses which is not yet found in any other Solanaceae host plants such as tomato and potato [6,18]. Although the *Pvr4* gene has been mapped to chromosome 10 of the pepper plant, it was not isolated, and subsequently the molecular mechanism of *Pvr4*-mediated resistance to PepMoV infection has not been elucidated [18]. Only a mutation of a RNA-dependent RNA polymerase (RdRp, also called NIb, hereafter NIb) area in PVY genome has been reported to confer virulence against *Pvr4*-bearing pepper plants [6]. However, a corresponding viral component that plays a role as an avirulence factor against *Pvr4* in pepper plants remains to be identified.

In this study, we screened all eleven proteins from PepMoV to identify the avirulence factor for the single dominant resistant gene, *Pvr4*, in CM334. Viral cistrons of PepMoV were cloned into an *in planta* expression vector for screening against *Pvr4*-segregating F2 populations derived from a cross between CM334 (*Pvr4*) and Jupiter (*pvr4*) cultivar. We revealed that NIbs from multiple potyviruses function as avirulence factors for Pvr4 in CM334.

## Materials and Methods

### Plant Materials

Six different *C*. *annuum* L. lines, including three resistance [CM334 (*Pvr4/Pvr4*), an F1 hybrid (*Pvr4/pvr4*), and a resistant homozygotic F2 (*Pvr4/Pvr4*) from a cross between CM334 and cv. Jupiter] and three susceptible lines [cv. ECW (*pvr4/pvr4*), cv. Jupiter (*pvr4/pvr4*), and a susceptible homozygotic F2 (*pvr4/pvr4*) from a cross between CM334 and cv. Jupiter] against PepMoV, were confirmed by viral inoculation and co-segregating DNA marker [18]. Briefly, to confirm resistance in pepper plants, we inoculated 4 to 6 weeks old leaves with PepMoV-GFP modified from PepMoV-Vb1 [19] and performed an enzyme-linked immunoassay (ELISA) to detect PepMoV according to the manufacturer’s protocol (Agdia, Elkhart, IN, USA). The genotypes of F1 and F2 lines were confirmed by *Pvr4*-linked co-segregating marker (PCAPS15) to distinguish *Pvr4* and *pvr4* genes [18]. Transient assays were performed with 4 to 6 week-old pepper plants. All pepper plants were grown in a growth chamber at 22–25°C with 60% relative humidity and a 14:10-hour light-dark cycle.

### Application of *Pvr4*-linked CAPS Marker for Identification of Pepper Genotype

**Fig 1:**
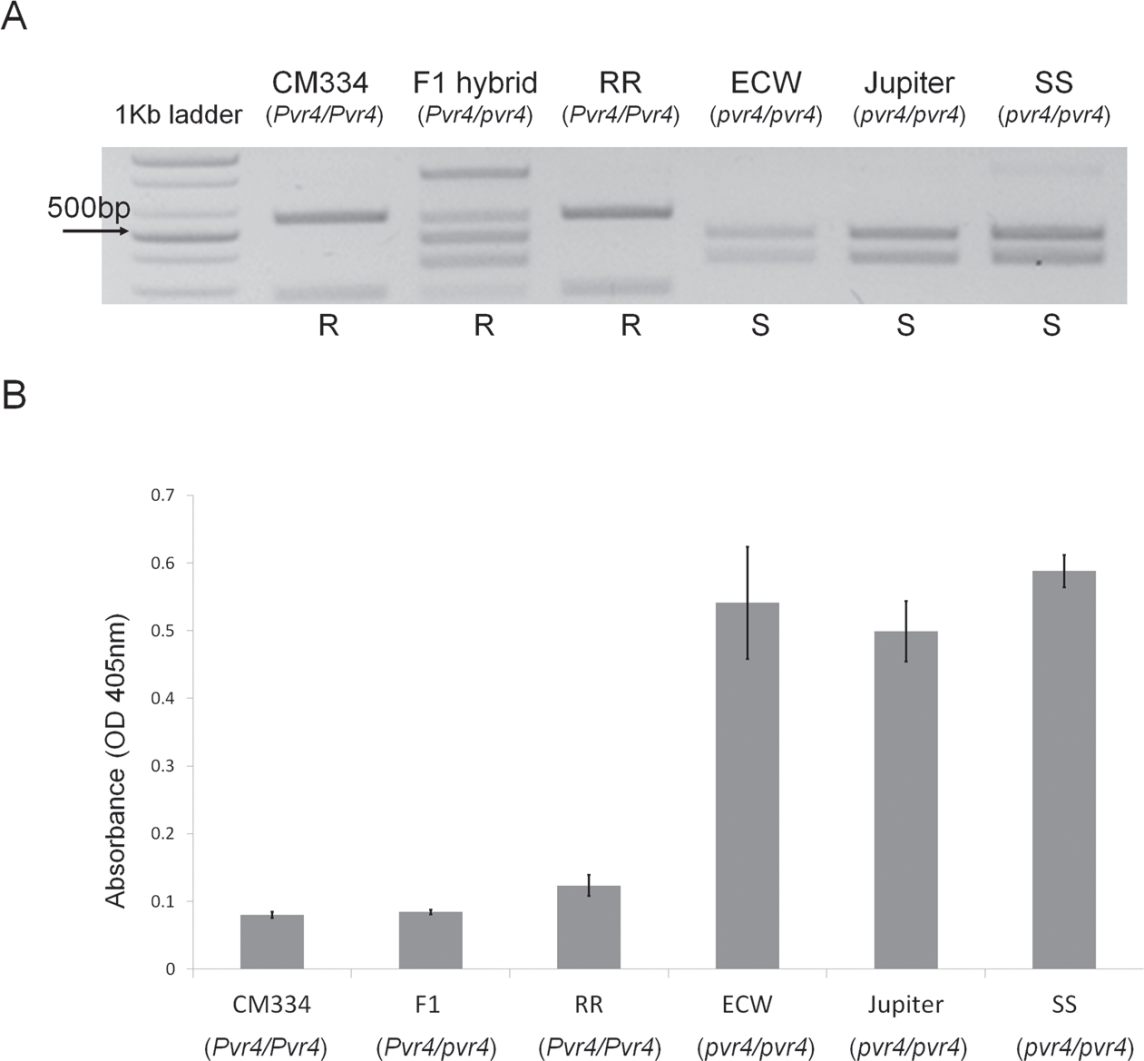
Genotypes and genotype-specific accumulation of PepMoV in pepper plants. (A) Identification of genotype in relation to *Pvr4* using the CAPS marker (PCAPS15). *Pvr4*-harboring pepper genotypes have 550- and 270-bp fragments, while *pvr4*-plants have 470- and 350-bp fragments. RR; a resistant homozygotic F2, SS; a susceptible homozygotic F2. Genotype of each plant is depicted under the cultivar name, and phenotypes of plants are also described under the images. R denotes resistant, and S denotes susceptible. (B) Detection of accumulated PepMoV by ELISA. Resistance against PepMoV was confirmed by ELISA with PepMoV antibody, which presents an accumulation of virus. Genotype of each plant is depicted under the cultivar name. Pepper leaves were sampled at 15 dpi. Error bars represent standard deviations. This result and subsequent figures show a representative experiment of three biological replicates.

For detection of *Pvr4*-linked markers, PCR products that were amplified with the marker primer were digested with XhoI. *Pvr4*-linked CAPS marker (PCAPS15) allows discernment of the *Pvr4* allele as *Pvr4*/*Pvr4*, *Pvr4*/*pvr4*, or *pvr4*/*pvr4* [18]. As shown in Fig. 1A, XhoI digestion of the PCR products generated 550- and 270-bp fragments for *Pvr4* and 470- and 350-bp fragments for *pvr4*.

### Cloning of Potyvirus Cistrons for *in planta* Expression

For cloning of PepMoV cistrons for *in planta* expression, specific primers to amplify each coding regions and the *NIb* from PepSMV (NC_008393) [20], PVY (EF026074.1) [21] and TEV (M11458.1) [17] were designed for use in the ligation-independent cloning (LIC) method by adding adapter sequences with: 5’- CGACGACAAGACCCT ATG (adaptor sequence) – viral coding region specific sequence – 3’ and 5’ – GAGGAGAAGAGCCCT TCA (adaptor sequence)—viral coding region specific sequence – 3’ [22,23]. *P3N-PIPO* cistron was generated by overlap PCR including a PIPO coding region in the GGAAAAAA motif to place the *PIPO* ORF in-frame with the N-terminal half of the P3 coding region [3,24–26]. For cloning of PepMoV cistrons for western blot, specific primers added HA tag (TACCCATACGACGTCCCAGACTACGCT) to amplify *NIb*, *CP* and *HC-Pro* were designed for use in the ligation-independent cloning (LIC) method by adding adapter sequences with: 5’ - GAGGAGAAGAGCCCT (adaptor sequence) TCA AGCGTAGTCTGGGACGTCGTATGGGTA– viral coding region specific sequence – 3’ in C-terminal region (S1 Table). As a control, Coat Protein (CP) coding regions from PepSMV and PVY-0 were designed for use in the ligation-independent cloning (LIC) method by adding adapter sequences. All amplified PCR products were cloned by LIC method into the pCAMBIA2300-LIC vector containing the CaMV 35S promoter and the NOS terminator cassette [22,23]. A total 15 fmol of purified PCR product was treated with T4 DNA polymerase (NEB) in reaction buffer containing 10 mM dATP at 22°C for 30min and 70°C for 20min for inactivation of T4 DNA polymerase. The pCAMBIA2300-LIC vector was digested with *PstI* and treated with T4 DNA polymerase with 10 mM dTTP. T4 DNA polymerase-treated PCR products and pCAMBIA2300-LIC vector were mixed and incubated at room temperature for 30 min [22]. The mixture was transformed into *E*. *coli* DH10b competent cells. The entire sequence of cloned cistrons was confirmed by DNA sequencing at the National Instrumentation Center for Environmental Management (NICEM, Seoul, Korea). Each cloned vector was transformed into *Agrobacterium tumefaciens* strain C58C1 for transient *in planta* expression assays [27].

### *In planta* Expression Assay in Pepper Plants

After transformation, the cultured cells were centrifuged and re-suspended in induction buffer (10 mM MgCl_2_, 10 mM MES pH 5.6, and 200 μM Acetosyringone), and cells were incubated at room temperature for 2 h before agro-infiltration. The concentration of *Agrobacterium* cells was adjusted to 0.5 at OD_600_, and then the cells were subjected to pressure infiltration using needleless syringe [28]. Empty vector and vector with necrosis-inducing protein (NIP) from *Phytophthora sojae* were infiltrated into one pepper leaf as a negative or positive control, respectively [29]. All experiments were performed as three biological replicates. Cell death on the leaves was observed at two or three days after *Agrobacterium* infiltration. Inoculated leaves were cleared in 100% ethanol to remove chlorophyll in order to visualize the cell death. Total RNA was extracted from pepper plant using TRIzol (Invitrogen, http://www.invitrogen.com/) according to the manufacturer’s instructions. First strand cDNA was synthesized using 3 μg total RNA with oligo (dT) and Superscript II reverse transcriptase (Invitrogen) for RT-PCR. Oligonucleotides used in RT-PCR were described in S1 Table.

### Immunodetection of PepMoV-encoded proteins

To confirm the *in planta* expression of viral proteins, we representatively decided to design three HA-tagging constructs out of eleven viral-encoded proteins. HA tag sequence was added at C-terminal of PepMoV NIb, CP and HC-Pro (See Material and methods, Cloning of Potyvirus Cistrons for *in planta* Expression). These constructs were transformed into *Agrobacterium* C58C1 and the cells were fully infiltrated into *N*. *benthamiana* leaves. Total protein was extracted from leaves of *N*. *benthamiana* with extraction buffer as described in Win *et al* [30] at 1 day and 2 days after infiltration of each construct. Protein concentrations were measured by Bradford assay (Thermo Scientific, Waltham, Massachusetts, United States), and equal amounts were loaded onto polyacrylamide gels. After transfer, western blot analysis was accomplished to detect protein expression by using an anti-HA antibody (Abcam, Cambridge, UK) and an anti-rabbit horseradish peroxidase conjugate (Abcam, Cambridge, UK).

## Results and Discussions

### Genotypes and PepMoV Accumulation in Pepper Plants

To confirm *Pvr4*-mediated resistance in pepper plants, we performed genotype screening by PCR with the PCAPS15 marker, and then utilized ELISA to detect PepMoV accumulation [18]. When the marker was applied in pepper, *Pvr4*-harboring pepper genotypes showed 550- and 270-bp fragments, while *Pvr4*-lacking (*pvr4*-) plant genotype showed 470- and 350-bp fragments. In our results, CM334, F1 hybrid and the resistant homozygotic F2 (RR) lines contained band patterns of *Pvr4*-harboring genotype, whereas the other peppers had band patterns of *Pvr4*-lacking genotype (Fig. 1A). Resistance against PepMoV could be confirmed by ELISA with a PepMoV antibody, which presents an accumulation of virus. Lower values (ELSIA value < 0.2) which were detected with CM334, F1 hybrid and the resistant homozygotic F2 lines represented that PepMoV replication was limited in those peppers. On the other hand, ECW, Jupiter and the susceptible homozygotic F2 (SS) lines showed higher values (ELSIA value > 0.4) (Fig. 1B). These results indicated that *Pvr4*-harboring plants successfully repressed the growth of PepMoV virus and that resistance phenotypes of pepper plants against PepMoV co-segregated with their genotypes. From these conclusions, we decided to use these pepper lines for screening the avirulence factor of potyviruses.

### Identification of NIb as the Avirulence Factor of PepMoV in *Pvr4*-bearing Pepper Plants

**Fig 2:**
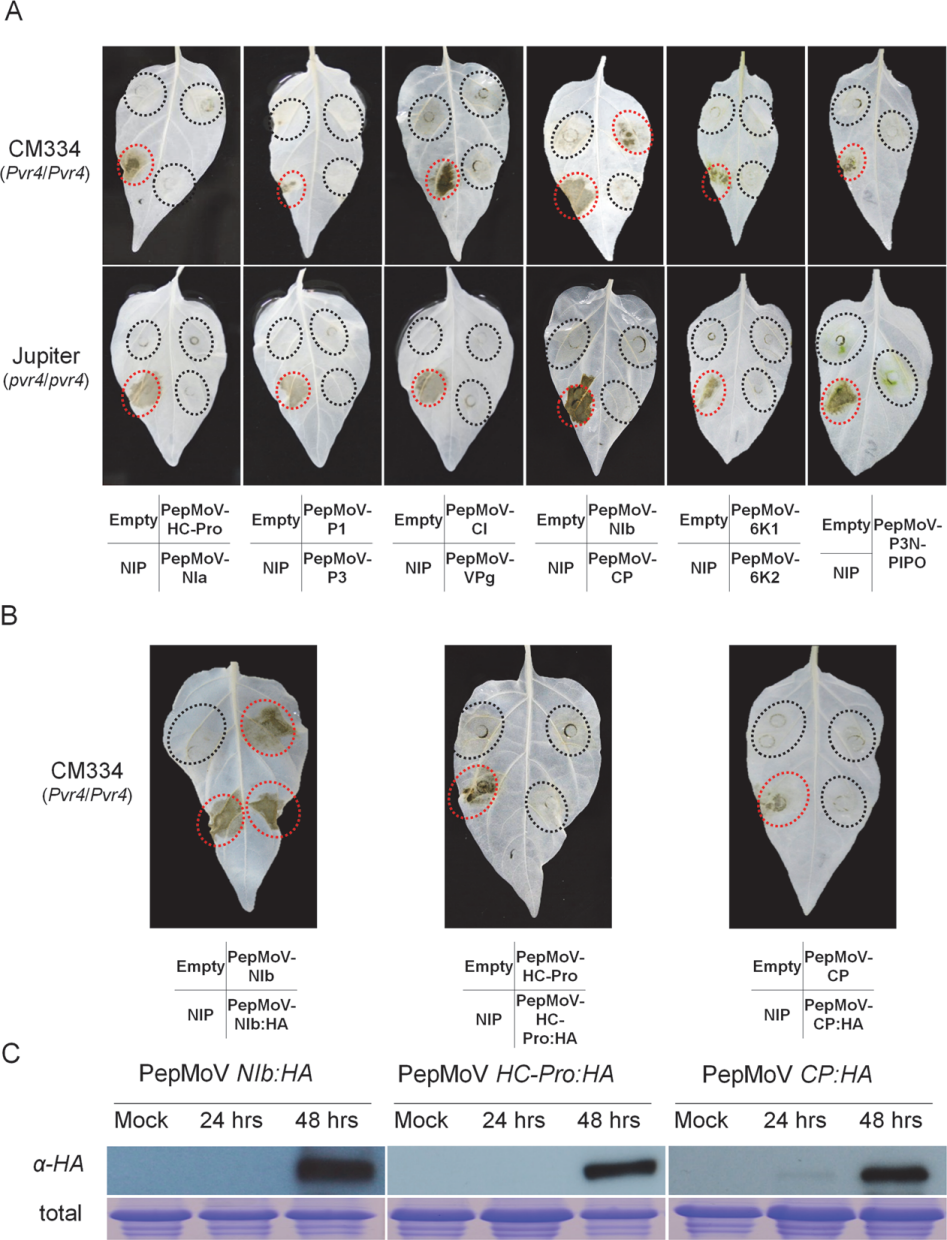
Identification of NIb as the HR-inducing avirulence factor against *Pvr4*-bearing pepper plants. (A) Transient expression of PepMoV viral proteins in CM334 and Jupiter. Eleven cistrons from PepMoV were infiltrated into CM334 and Jupiter. At 3 dpi, leaves were cleared with 100% ethanol to remove chlorophylls in order to visualize the cell death. For this and subsequent experiments, Empty vector and vector with necrosis-inducing protein (NIP) from *P*. *sojae* were infiltrated as a negative or positive control, respectively. Regions of infiltration were marked with ovals and the area of cell death was marked as red. Inoculated viral cistrons were depicted under panels. (B) Transient expression of HC-Pro:HA, CP:HA and NIb:HA in CM334. Plant responses with HA-tagged proteins were tested in *Pvr4*-harboring plants (CM334). Inoculated viral cistrons were depicted under panels. (C) Expression of PepMoV NIb:HA, CP:HA and HC-Pro:HA proteins in *N*. *benthamiana* leaves. 5-week-old tobacco leaves were collected at 24hpi and 48hpi. Untreated leaves were used as mock for negative controls. Each protein was immunodetected by using anti-HA antibody. Coomassie blue–stained total proteins were shown as loading controls.

To identify the avirulence factor of PepMoV, we performed *in planta* expression analyses with eleven viral proteins of PepMoV in pepper plants (Table 1). First, PepMoV coding regions were dissected and cloned into the pC2300-LIC binary vector with a 35S promoter [1,2]. For *in planta* expression analyses, each clone was infiltrated in all six pepper cultivars, respectively. As results, HR-like cell death was observed only in the PepMoV NIb-expressing leaves in a genotype-specific manner. However, the HR-like cell death was absent when other viral cistrons were infiltrated (Fig. 2A and S1 Fig.)

**Table 1:** PepMoV cistrons used in this study.

Name of cistron	Size (bp)	Function	References
P1	861	serine protease	[32]
HC-Pro	1368	helper-component protease	[10]
P3	1083	potyviral membrane protein	[3,33]
6K1	156	unknown	-
CI	1902	cylindrical inclusion	[34]
6K2	156	potyviral membrane protein	[33]
VPg	564	viral protein genome-linked	[35]
NIa (Pro)	738	nuclear inclusion A	[36]
NIb	1557	RNA dependent RNA polymerase	[6,37]
CP	819	coat protein	[38]
P3N-PIPO	771	cell-to-cell movement	[3,24]

To test whether each clone from PepMoV interacts with Pvr4 at the protein level, we picked three clones, NIb, HC-Pro, and CP from PepMoV and generated HA-tagged constructs (PepMoV NIb:HA, PepMoV HC-Pro:HA and PepMoV CP:HA). Each protein expression was detected by western blot experiments using anti-HA at 24 and 48 hours after infiltration in *N*. *benthamiana* (Fig. 2C). To verify that these proteins still have their activity in *Pvr4*-mediated resistance, we performed *in planta* expression of these HA-tagged proteins in CM334 and also observed HR-like cell death with PepMoV NIb:HA regardless of whether the HA tag was present or not. Over-expression of other cistrons such as PepMoV HC-Pro and PepMoV CP did not induce HR-like cell death in CM334 (Fig. 2B). This results suggested that the PepMoV NIb protein works as the avirulence factor in *Pvr4*-containing CM334.

**Fig 3:**
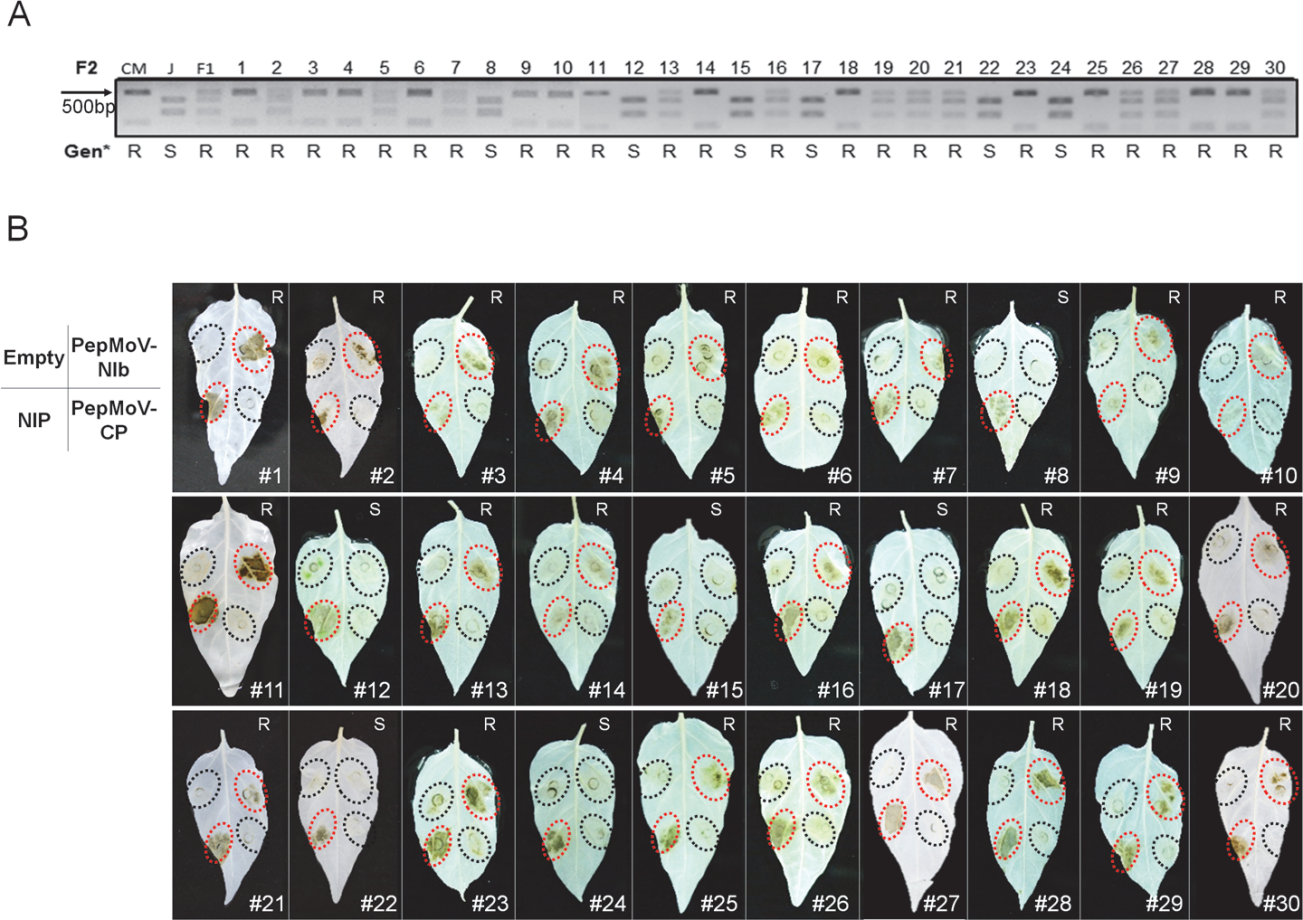
Correlation of genotypes and cell death phenotype of *Pvr4* against NIb in the F2 population. (A) Identification of genotype in relation to *Pvr4* using the CAPS marker (PCAPS15). Thirty plants of the F2 generation were tested to identify their genotypes. Genotypes of plants (Gen*) are described under the images as R (resistant) or S (susceptible). (B) Response of the F2 population plants derived from Jupiter and CM334 to PepMoV proteins, NIb and CP. Thirty progenies of the F2 generation were tested to verify whether *Pvr4*-harboring plants show HR in response to PepMoV NIb. The F2 lines which showed HR cell death as well as *Pvr4* genotypes were marked as R. S represents the F2 lines which did not show HR cell death and were confirmed as *pvr4*-plants. Inoculated viral cistrons were depicted at the left of panel.

To investigate the correlation of NIb-induced cell death with *Pvr4* gene in pepper, we also examined the phenotypes of the F2 population derived from CM334 and Jupiter by transient expression of PepMoV *NIb*. The genotypes of the F2 segregating progenies of the cross between CM334 and Jupiter were clarified by the PCAPS15 marker analysis (Fig. 3A). All *Pvr4*-bearing plants showed HR cell death while none of *pvr4*-plants show HR cell death (Fig. 3B). This results implied that HR-like cell death phenotype induced by PepMoV NIb is related to *Pvr4*.

In a previous study, it was suggested that an untranslatable RNA sequence of the Cymbidium Ringspot Virus (CymRSV) CP might be a HR inducing elicitor in *Datura stramonium* [31]. To confirm the *NIb* RNA itself does not cause HR-like cell death, we generated the frame-shifted mutant of *NIb* (PepMoV-ΔNIb) and transiently expressed in the F2 populations derived from Jupiter and CM334. Expression of *PepMoV NIb* and *PepMoV-ΔNIb* were confirmed in pepper leaves tested by RT-PCR (S2 Fig.). The NIb mutant did not induce HR-like cell death phenotype in any tested pepper plants while the in-frame NIb construct showed HR cell death (S2 Fig.). This result indicated that HR-like cell death was not induced by *NIb* RNA in resistant pepper plants, but by NIb protein. Taken together, these results clearly demonstrate that the PepMoV NIb protein is the avirulence factor for *Pvr4* in pepper plants.

### NIb proteins of other Potyviruses as Avirulence Factors in *Pvr4*-mediated Resistance

**Fig 4:**
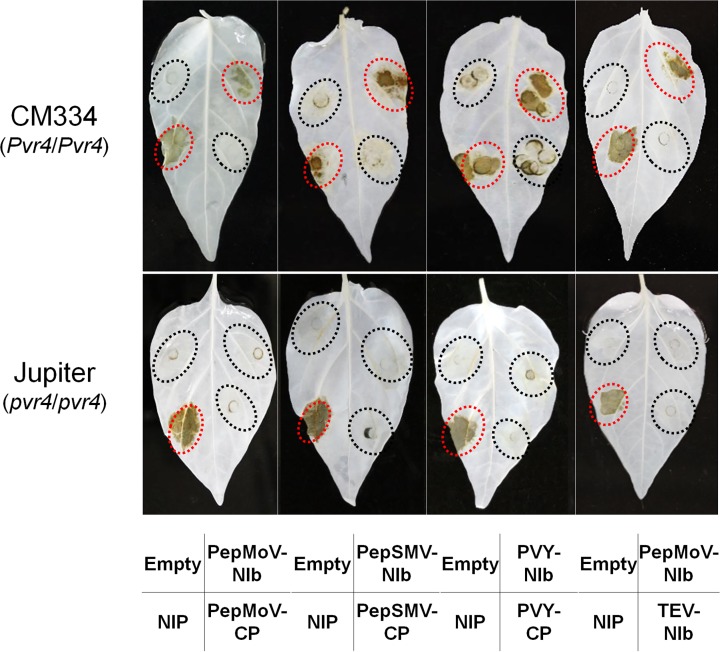
Confirmation of NIb as the HR-inducing avirulence factor against *Pvr4*-bearing pepper plants. *In planta* expressions of NIbs from four potyviruses were performed in CM334 and Jupiter, respectively.

To test whether NIb proteins from other potyviruses function as avirulence factors, we cloned NIb coding regions from potyviruses PepSMV and PVY into the pCAMBIA2300-LIC vector and examined *in planta* expression assays with pepper plants. When each *NIb* cistron was transiently expressed in each pepper plants, HR-like cell death was observed only in *Pvr4*-containing plants (CM334, the F1 hybrid, and the resistant homozygotic F2) (Fig. 4 and S3 Fig.). These results indicate that NIbs of PepSMV and PVY also function as Pvr4 interactants in the plant immune system.

Since TEV is a virulent potyvirus to *Pvr4*-bearing pepper plants, we tested whether TEV NIb interacts with Pvr4 and subsequently causes cell death. Thus, TEV NIb coding region was cloned into pC2300-LIC vector and *in planta* expressed in leaves of CM334 and Jupiter. However, HR-like cell death was not observed in any pepper leaves when the clone was infiltrated (Fig. 4). Taken together, although TEV has NIb like other potyviruses, TEV NIb could not induce HR-like cell death and additionally TEV shows virulence in *Pvr4*-bearing pepper plants (Table 2). The reason why TEV NIb does not cause HR-like cell death is likely that it has a difference in structure compared to other three potyviruses NIbs. In previous study, TEV diverged from other three potyviruses in phylogenetic tree when parts of these nucleotide sequences were compared [17]. Furthermore, when we compared the identity of NIb proteins among four potyviruses, TEV NIb had 61% identity compared with PepMoV, PepSMV and PVY, while three potyviruses have at least 76% identity. This result infers that TEV NIb, which has lower identity to other potyviruses NIbs, may not be recognized by Pvr4.

**Table 2:** Resistance and HR induced NIb of potyviruses in *Pvr4*-harboring pepper plants.

Potyvirus species	NIb-induced HR	Virus resistance	
		Phenotype	References
PepMoV (DQ631638)	+	R	In this study, [18,19]
PepSMV (NC_008393)	+	R	[18,20]
PVY (EF026074)	+	R	[18,39]
TEV (M11458)	−	S	[18,39]

HR, hypersensitive response. R, resistant; S, susceptible.

PepMoV; *Pepper mottle virus*, PepSMV; *Pepper severe mosaic virus*, PVY; *Potato virus Y*, TEV; *Tobacco etch virus*.

+, HR induced;

−, HR not induced.

In sum, the high similarity of NIb protein sequences in avirulent potyviruses might be important for these proteins to function as avirulence factors. Subsequently, this would mediate a broad-spectrum stable resistance for *Pvr4*-bearing pepper plants.

## Conclusion

In this study, we demonstrated that NIb proteins of three potyviruses are common avirulence factors for *Pvr4*-mediated resistance in pepper plants. These results may provide an efficient tool for the isolation of the broad-spectrum potyvirus resistance gene *Pvr4* from pepper, as well as for studying potyvirus resistance mechanisms in plants.

## Supporting Information

S1 Fig Identification of NIb as the HR-inducing avirulence factor against Pvr4-bearing pepper plants. Transient expression of PepMoV viral proteins in the resistant homozygotic F2 (RR), F1 hybrid, ECW and the susceptible homozygotic F2 (SS). Eleven cistrons from PepMoV were infiltrated into four pepper cultivars.
(TIF)

S2 Fig Verification of NIb-encoded protein as the avirulence factor against *Pvr4*-bearing pepper plants. (A) Response of five pepper cultivars after *in planta* expression of *NIb* or frame-shifted *NIb* mutant clone of PepMoV at 2–3 dpi. (B) RT-PCR of transient overexpressed PepMoV *NIb* and -*ΔNIb*. Pepper leaves were sampled at 0, 12, 18, 24 and 48 hours after transient overexpression. As a control, *actin* was used.
(TIF)

S3 Fig Confirmation of NIb as the HR-inducing avirulence factor against *Pvr4*-bearing pepper plants. *In planta* expressions of NIbs from four potyviruses were performed in four cultivars, respectively.
(TIF)

S1 Table Primer sequences used in this study. (XLSX)
