# Supplementary figures and images for: RNA-Dependent RNA Polymerase (NIb) of the Potyviruses Is an Avirulence Factor for the Broad-Spectrum Resistance Gene Pvr4 in Capsicum annuum cv. CM334
Source: PLoS One. 2015 Mar 11;10(3):e0119639. doi: 10.1371/journal.pone.0119639 (PMC4356556; doi:10.1371/journal.pone.0119639)

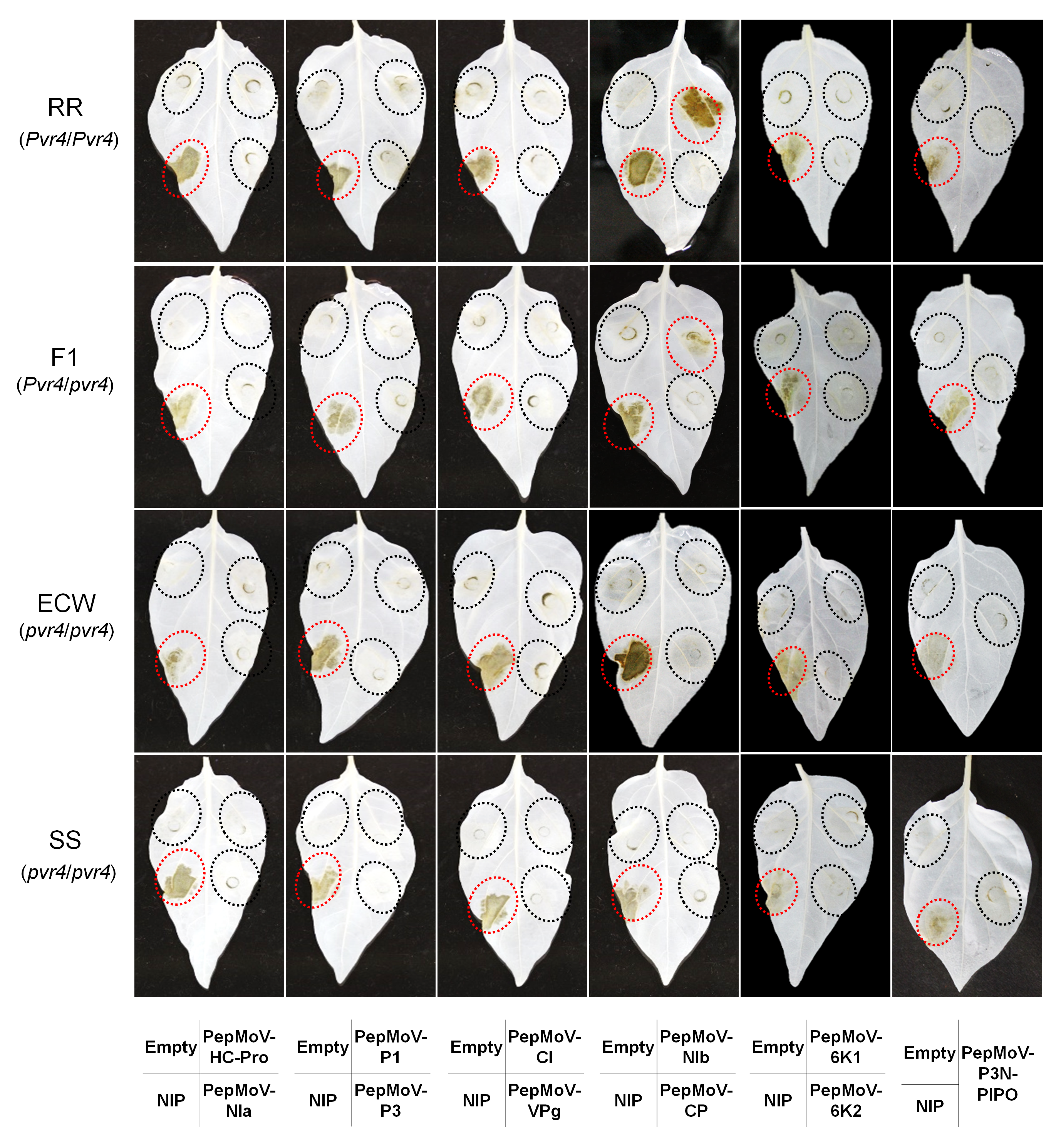

Supplement: S1 Fig — Transient expression of PepMoV viral proteins in the resistant homozygotic F2 (RR), F1 hybrid, ECW and the susceptible homozygotic F2 (SS). Eleven cistrons from PepMoV were infiltrated into four pepper cultivars. (TIF) [file pone.0119639.s001.tif]

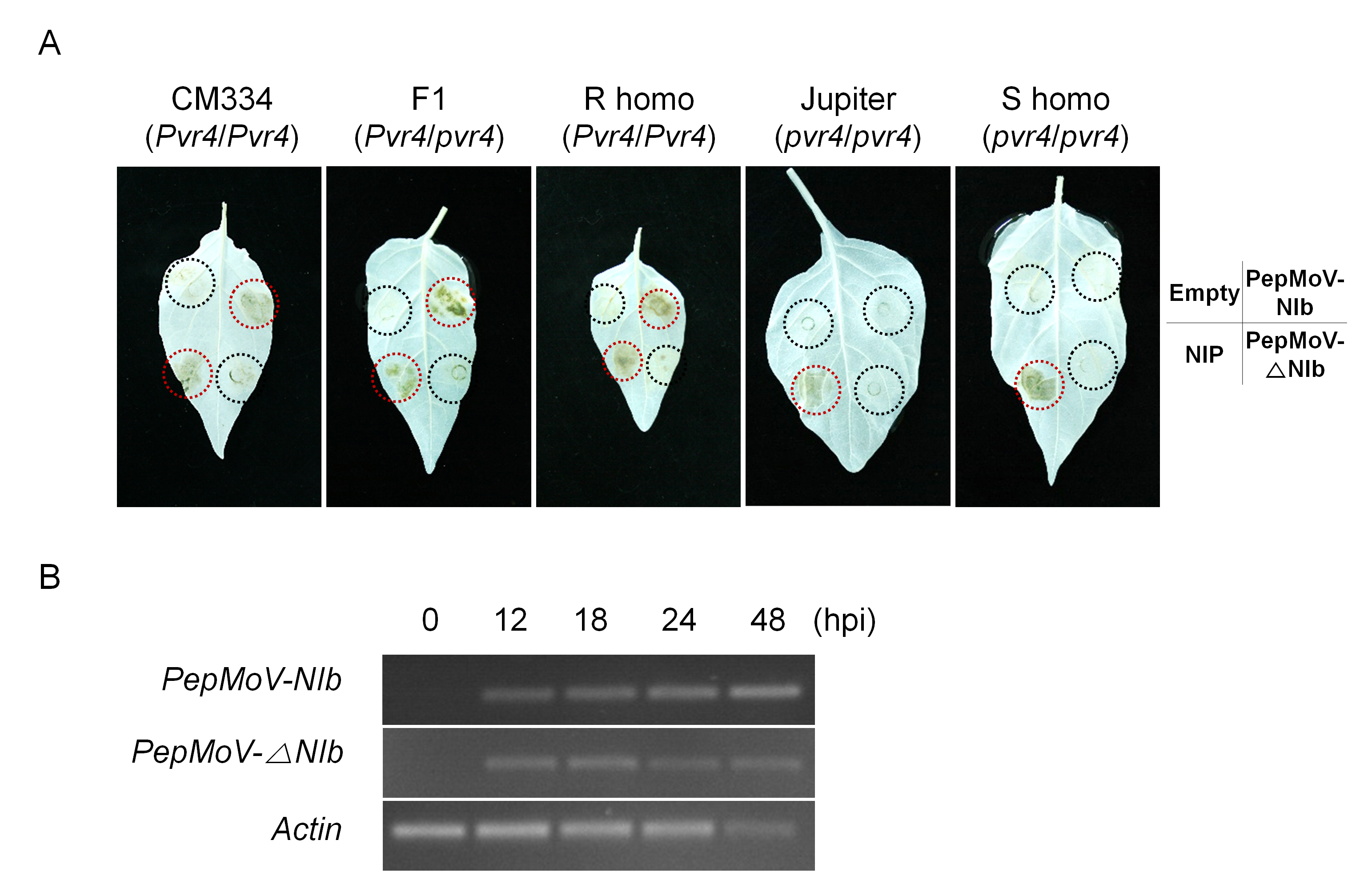

Supplement: S2 Fig — (A) Response of five pepper cultivars after in planta expression of NIb or frame-shifted NIb mutant clone of PepMoV at 2–3 dpi. (B) RT-PCR of transient overexpressed PepMoV NIb and -ΔNIb. Pepper leaves were sampled at 0, 12, 18, 24 and 48 hours after transient overexpression. As a control, actin was used. (TIF) [file pone.0119639.s002.tif]

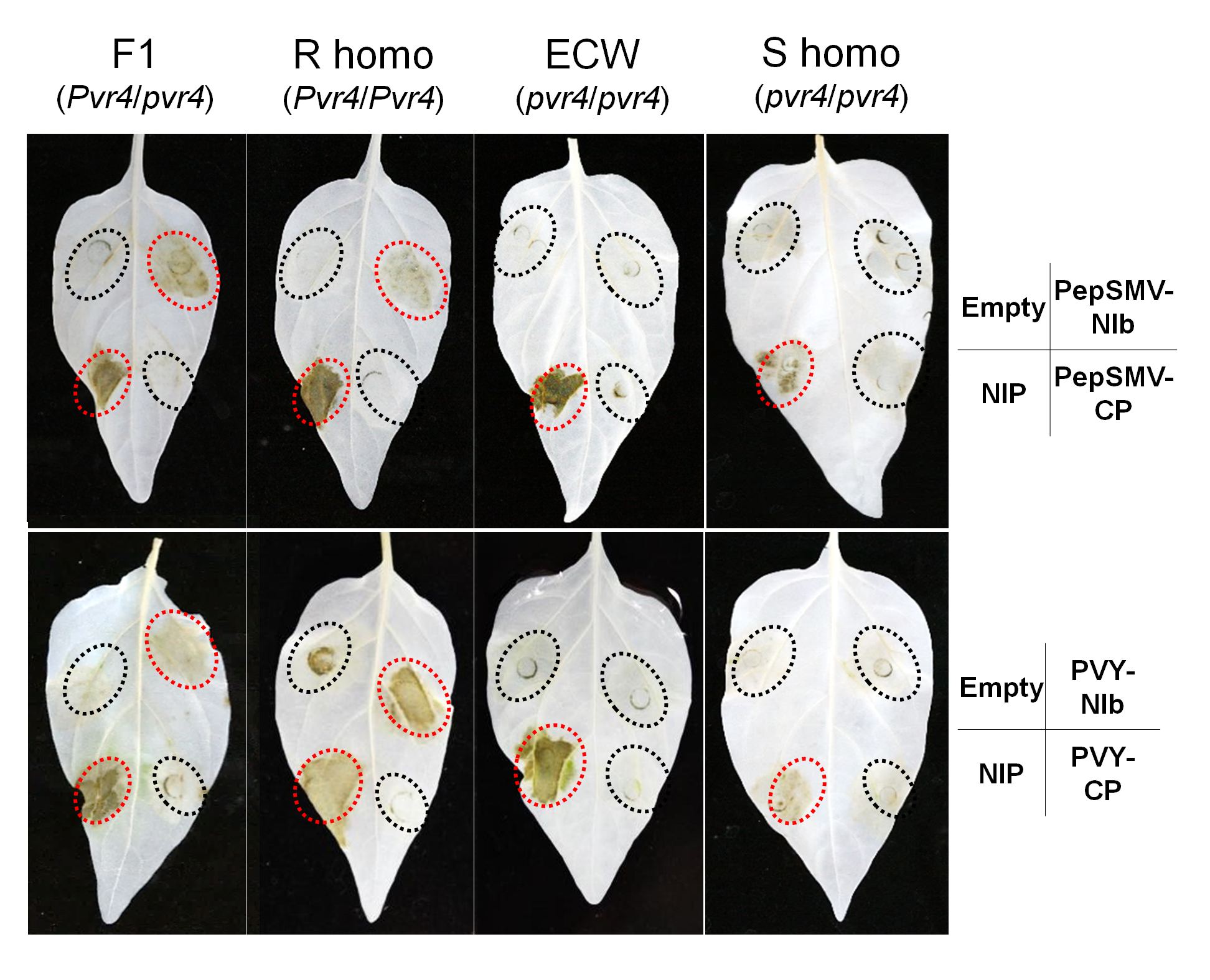

Supplement: S3 Fig — In planta expressions of NIbs from four potyviruses were performed in four cultivars, respectively. (TIF) [file pone.0119639.s003.tif]
